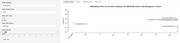# Genetic Colocalization of Expression Quantitative Trait Loci (eQTL) Mapping and GWAS in a multiethnic brain bank: An Insight into ancestry‐specific Regulatory Architecture in Alzheimer's disease

**DOI:** 10.1002/alz70855_107199

**Published:** 2025-12-25

**Authors:** Vivek Ruhela, Basilio Cieza, Richard Mayeux, Dolly Reyes‐Dumeyer, Andrew F. Teich, Giuseppe Tosto

**Affiliations:** ^1^ Columbia University, New York, NY, USA; ^2^ Departments of Neurology, Psychiatry, and Epidemiology, Gertrude H. Sergievsky Center, The Taub Institute for Research on Alzheimer's Disease and the Aging Brain, Vagelos College of Physicians and Surgeons, Columbia University, New York, NY, USA; ^3^ Gertrude H. Sergievsky Center, Vagelos College of Physicians & Surgeons, Columbia University, New York, NY, USA; ^4^ Department of Neurology, College of Physicians and Surgeons, Columbia University, and the New York Presbyterian Hospital, New York, NY, USA

## Abstract

**Background:**

Expression quantitative trait loci (eQTL) have been identified using tissue or cell samples from diverse human populations, enhancing our understanding of gene expression regulation in the context of complex diseases, such as Alzheimer disease (AD). However, few studies have attempted to identify eQTL across multiple ethnic groups. We employed prefrontal cortical brain samples from the New York Brain Bank at Columbia University.

**Method:**

We analyzed RNA‐Seq data from 32 Hispanics and 263 Non‐Hispanic White (NHW) prefrontal cortical brain samples. Stratified cis‐ and trans‐eQTL analyses were performed using TensorQTL, while GWAS analysis was conducted on common variants (MAF > 5%) employing a linear mixed model. To assess genetic colocalization in Hispanics and NHW brains, we applied eCAVIAR to estimate the likelihood of variants being causal, integrating both eQTL and GWAS signals.

**Result:**

In the colocalization of cis‐eQTL and GWAS signals for Hispanics, we observed a high posterior probability (posterior prob = 0.9947) for the upstream gene variant rs755980338 on chromosome 11, which locally regulates *EHD1* gene (GWAS *p*‐value = 0.009, cis‐eQTL *p*‐value = 0.0041). Similarly, in trans‐eQTL analysis of NHW brains, we identified strong colocalization for the upstream gene variant rs7840855 on chromosome 8 (posterior prob = 0.7, GWAS *p*‐value = 3.4e‐06), regulating both *TMEM68* (trans‐eQTL *p*‐value = 5.36e‐06) and *DEFA10P* (trans‐eQTL *p*‐value = 9.52e‐06).

**Conclusion:**

This study highlights the importance of population‐stratified eQTL and colocalization analysis in identifying genetic regulatory mechanisms underlying complex diseases such as AD. The high colocalization probabilities indicate shared causal variants, supporting the functional relevance of these genes in AD risk. Our findings suggest that *EHD1, TMEM68*, and *DEFA10P* may play an ancestry‐specific role in AD. For example, *EHD1* gene is involved in neuronal BACE1 transcytosis which is highly relevant for AD. Similarly, *TMEM68* was colocalized with chromosome 8 in brain prefrontal cortex and hypothalamus in GTEx panel. These insights contribute to a better understanding of AD genetic architecture, with potential implications for precision medicine approaches across populations. Results can be visualized via our newly‐developed *BRAINscape* (Figure 1) an interactive Shiny‐based interface for seamless data visualization to facilitate the integrative analysis of multi‐omics data.